# Supplementary material for: Complexome profiling of the Chlamydomonas psb28 mutant reveals TEF5 as an early PSII assembly factor
Source: Plant Cell. 2025 Mar 17;37(6):koaf055. doi: 10.1093/plcell/koaf055 (PMC12164590; doi:10.1093/plcell/koaf055)
Supplement: koaf055_Supplementary_Data [file koaf055_supplementary_data.zip › psb28_interactive.zip!/plots/intensity/qualityPlots/peptides/CPLD62_Cre12.g538700.t1.2.html]

### Cre12.g538700.t1.2

Trivial name: CPLD62  
SeqCoverage [median]: 11.00  
Mass: 93.49 kDa  
  
 

| Sequence | MissedCleavage | Mass [Da] | wtIntensity | muIntensity | Score |
| --- | --- | --- | --- | --- | --- |
| EAVNFLTR | 0 | 948.50 | 0.00 | 724.55 | 64.27 |
| GPSAEEDKALK | 1 | 1143.58 | 600.66 | 629.65 | 77.38 |
| LAAEIPDMR | 0 | 1014.52 | 2744.89 | 4653.05 | 101.54 |
| LLGSDVNTGVR | 0 | 1129.61 | 0.00 | 1133.29 | 72.64 |
| LTTLVETR | 0 | 931.53 | 0.00 | 366.79 | 103.83 |
| VAGEIIANNIKPALK | 1 | 1549.92 | 0.00 | 3026.15 | 90.93 |
| VGVADQTVFGGQGFSLTK | 0 | 1809.93 | 0.00 | 843.68 | 85.85 |
| YEELER | 0 | 837.39 | 0.00 | 636.77 | 66.99 |
|  |  |  | 3345.6 | 12013.9 |  |
